# Stability of dietary patterns assessed with reduced rank regression; the Zutphen Elderly Study

**DOI:** 10.1186/1475-2891-13-30

**Published:** 2014-04-01

**Authors:** Nicole Jankovic, Martinette T Steppel, Ellen Kampman, Lisette CPGM de Groot, Hendriek C Boshuizen, Sabita S Soedamah-Muthu, Daan Kromhout, Edith JM Feskens

**Affiliations:** 1 Division of Human Nutrition, Wageningen University, PO Box 8129, 6700 EV Wageningen, The Netherlands

**Keywords:** Dietary pattern, Stability, Elderly, Exploratory reduced rank regression analyses, Confirmatory reduced rank regression analyses

## Abstract

**Background:**

Reduced rank regression (RRR) combines exploratory analysis with a-priori knowledge by including risk factors in the model. Dietary patterns, derived from RRR analysis, can be interpreted by the chosen risk factor profile and give an indication of positive or adverse health effects for a specific disease. Our aim was to assess the stability of dietary patterns derived by RRR over time.

**Methods:**

We used data from 467 men, aged 64–85 years, participating in the 1985 and 1990 examination rounds of the Zutphen Elderly Study. Backwards regression on risk factors and food groups was applied prior to the RRR analysis to exclude food groups with low predictability (from 36 to 19 food groups) for the chosen risk factor profile. For the final RRR analysis, dietary intake data from 19 food groups as predictor variables and 6 established risk factors for cardiovascular diseases (body mass index, systolic and diastolic blood pressure, high density lipoprotein and total cholesterol levels, and uric acid) were used.

**Results:**

Three RRR dietary patterns were derived for both examination years: a “(low in) cereal fibre pattern”, an “alcohol pattern” and an “inconsistent pattern”. The “(low in) cereal fibre pattern” was most stable over time, with a correlation coefficient of 0.47 (95% CI: 0.38-0.53) between 1985 and 1990 measurements.

**Conclusion:**

Dietary patterns as measured by RRR, after backwards regression, are reasonably stable over a period of five years. Thus, RRR appears to be an attractive method to measure long-term dietary exposure for nutritional epidemiological studies, with one dietary measurement at baseline.

## Background

Two main approaches exist to derive dietary patterns: the a-priori and the a-posteriori approach. While a-priori defined dietary indices give an indication of a populations diet quality, the a-posteriori approach uses available dietary data to describe a populations diet. Such data reduction methods are principal component (PCA) or factor analysis and cluster analysis. Factors derived from these analyses represent actual dietary patterns of the studied population [1]. Both methods have widely been applied in nutritional epidemiology, and the stability of dietary patterns derived from PCA and factor analysis was examined previously [2-6].

Another a-posteriori method to study dietary patterns is called reduced rank regression (RRR) and was introduced to nutritional epidemiology by Hoffmann *et al*. [7]. RRR finds dietary patterns that are potentially relevant for a disease by using a-priori knowledge, for example on biological risk factors or nutrients relevant for the disease of interest. The initial idea of analysing food groups in relation to risk factors, was to explain, describe and interpret diet-disease relationships based on changes in the chosen risk factors [8,9]. In contrast to PCA and factor analysis, RRR does not describe naturally occurring patterns of the population under study but explain variation in biologically important risk factors [9]. Previously, RRR has been used to derive dietary patterns associated with risk factors from baseline data for the analysis of chronic diseases and for tracking dietary patterns in children [10]. The stability of RRR patterns over time in elderly participants remain unknown [11,12]. Cohort studies often lack information on repeated measures over time and need to rely on baseline measurements, assuming stability of long-term exposure. Therefore, we assessed the long-term stability of dietary patterns, derived from RRR, in elderly men on the population level. This analysis will add to the knowledge and possible implications of RRR analysis in nutritional epidemiology.

## Methods

### Study population

The Zutphen Elderly Study started in 1985 to collect longitudinal population-based data on risk factors of cardiovascular diseases and health in elderly men living in the town of Zutphen, in the eastern part of The Netherlands. At baseline, 939 elderly Dutch men, aged 64–85 years (response rate 74%), participated in this study. Every five years from 1985 until 2000, the subjects’ dietary intake and cardiovascular disease risk factors were measured [13,14]. Excluding participants with missing data on dietary intake or response variables, reduced the sample from 939 to 763 participants. Five years after baseline, measurements were collected from 560 elderly men (response rate 78%). Additional exclusion of participants with missing information at follow-up resulted in a sample of 467 men eligible for further analyses.

This study was conducted according to the guidelines laid down in the Declaration of Helsinki and all procedures involving human subjects were approved by the Medical Ethics Committee of the Leiden University, The Netherlands in 1985 and 1990. Written informed consent was obtained from all participants.

### Assessment of dietary intake

The usual dietary intake of the last 2 to 4 weeks was assessed at the home of the participant by dieticians, applying the cross-check dietary history method [15], which was adapted to the Dutch situation. The dietary survey took place between March and June in 1985 and 1990, respectively. If possible, the partner/housemate who usually cooked the meals was present during the interview which consisted of two parts. For the first check, the dietician interviewed the participant about his usual food intake on weekdays and weekends. For the second check, the dietician quantified the foods bought per week and compared these values with the participants report. Both sources of information were used to estimate the participant’s usual food and alcohol consumption, energy and nutrient intake. Consumed foods were encoded by the dieticians, according to the Uniform Food Encoding System developed in the Netherlands [16]. After the coding, the foods were categorized into 36 food groups. Prevalent chronic diseases [13], were assessed by questionnaire information and confirmed by letters from general practitioners.

### Collection of response variables

According to a standardized protocol, height and weight were measured by a physician. Results were rounded to the nearest millimeter. Weight was recorded to the nearest 0.5 kg [14]. Body mass index (BMI) was calculated by dividing weight in kilograms by the height in meters^2^ (kg/m^2^). Systolic and diastolic (Korotkoff phase five) blood pressure were measured while participants were in supine position. Blood pressure measurements were taken twice at the end of the physical examination using a random-zero sphygmomanometer (Hawksley & Sons Ltd, West Sussex, United Kingdom) [14,17]. The mean value of the repeated measurements was used in the analyses. Non-fasting venous blood samples were used to determine total and high-density lipoprotein (HDL) cholesterol levels in the standardized Lipid Laboratory of our Division [18]. Uric acid was analysed by a standard procedure of an autoanalyser at the Central Clinical and Chemical Laboratory of the University Hospital of Leiden, The Netherlands (SMAC, Technicon).

### Statistical analyses

All statistical analyses were performed using SAS software, version 9.2 (SAS Institute Inc., Cary, North Carolina, United States) and a two-sided p-value < 0.05 was considered statistically significant.

To assess the stability of dietary intake over a period of five years, median food group intakes in grams per day were compared between baseline and five years of follow-up. Correlation coefficients were calculated to examine the ability to rank participants food group intake similarly over time. Due to the skewed distribution of food groups, Spearman correlations were used.

We started the RRR dietary pattern analyses, including six CVD risk factors as response variables: BMI, systolic and diastolic blood pressure, serum total, HDL cholesterol, and uric acid. The risk factor selection was based on prior knowledge [19-22] and the chosen risk factors were also applied previously in studies for the purpose of RRR analysis [23,24].

Random sample cross-validation and subsequent Van der Voet’s test as previously applied by Heroux *et al*. [23], were used, to define the number of dietary patterns, that best predict response variables and exclude chance findings of correlations. Cross-validation was performed on initially, 36 defined food groups and six risk factors. Both methods are described in detail elsewhere [25,26]. In short, random sample cross-validation forms 1000 random test sets of the initial dataset in which the RRR analyses is performed. The predictive power of the dietary patterns derived in each of the test sets, is summarized as the predicted residual sum of squares (PRESS). Based on the PRESS estimates, the Van der Voet’s test identifies the optimal number of dietary patterns. Each additional derived pattern would not contribute significantly to the explained variation in risk factors. In the initial analyses, the Van der Voet’s test indicated that no dietary pattern, based on 36 a-priori defined food groups, was able to predict the six response variables sufficiently. Derived patterns were strongly influenced by chance findings. Therefore, we reduced the number of food groups by applying backwards regression on the baseline and follow-up single response variables and corresponding food group data. Food groups that were important for baseline and for more than two response variables (p = 0.05) either in 1985 or 1990 were included in the model. Food groups not contributing to the explained variation in response variables would be eliminated. Finally, we ran exploratory RRR analysis independently for 1985 and 1990 and assigned a z-score per individual for each of the derived patterns for both study years [24]. The stability of dietary patterns derived from RRR was examined by comparing the food groups with a high weight (> 0.10 or < - 0.10) in each pattern at baseline and follow-up, the direction of the weights and the ability of the patterns to classify individuals similarly over time. As RRR z-scores are normally distributed, Pearson correlation coefficients were calculated between baseline and follow-up dietary patterns. Labelling of the derived food patterns was performed by using the highest positive or inverse food group weight of each dietary pattern at baseline and follow-up.

We ran a confirmatory RRR analysis to differentiate the influence of food group consumption and changes in biomarker profile over time, on the stability of dietary patterns. With this approach food group- and risk factor weights were fixed, which is different from the exploratory analysis where weights were first established through RRR [5,24]. Confirmatory factor scores were calculated by multiplying the fixed food group factor weights derived in 1985, with the standardized dietary intake data of 1990 and vice versa. For the assessment of diet changes, Pearson correlation coefficients were calculated between exploratory and confirmatory dietary pattern scores using dietary intake of the other year. The influence of biomarkers can partially (as RRR weights are influenced by food groups and response variables) be examined by the correlation coefficient between exploratory and confirmatory dietary pattern scores using dietary intake of the same year. For reasons of simplicity, we will only discuss correlation coefficients for exploratory pattern 1985 with confirmatory 1990 (change in diet) and confirmatory 1985 (change in weights).

The following sensitivity analysis were performed to assess the influence on stability: food groups were energy-adjusted prior to the RRR analysis using the residual method [27]; BMI was excluded from the response set to ascertain that dietary patterns derived are not solely BMI driven; separate RRR analyses were performed in participants without chronic diseases (myocardial infarction, stroke, diabetes or cancer) at baseline, as epidemiological studies often exclude participants with prevalent diseases; and finally, to assess the influence of reducing the study sample on the derived dietary patterns, RRR was additionally applied to the full study population of 763 participants in 1985 (describing the sample prior exclusion of participants dying between 1985 and 1990). The correlation coefficient between the exploratory RRR score derived in the full study population at baseline and reduced sample of 1985, was calculated for 467 participants.

## Results

Table 1 describes the characteristics of the study population which included 467 men (aged 64–85 years at baseline). Substantial changes between baseline and follow-up were observed for several CVD risk factors. Mean diastolic blood pressure decreased by 3.8 mmHg and energy intake decreased by 844 kJ (correlation coefficients between response variables of 1985 and 1990 are presented in Additional file 1: Table S1). The prevalence of chronic diseases (myocardial infarction, stroke, diabetes or cancer) increased with at least four percentage points for each disease.

**Table 1:** Demographic and lifestyle characteristics of elderly men (N = 467), aged 64–85 years, participating in the Zutphen Elderly Study in 1985 and 1990

**Characteristics**	**1985**	**1990**
BMI (kg/m^2^)^*^	25.7 ± 3.0	25.5 ± 3.2
Systolic blood pressure (mmHg)^*^	150.0 ± 20.2	149.4 ± 21.4
Diastolic blood pressure (mmHg)^*^	85.5 ± 11.1	81.7 ± 11.8
Total-cholesterol (mmol/l)^*^	6.13 ± 1.07	6.07 ± 1.13
HDL-cholesterol (mmol/l)^*^	1.11 ± 0.26	1.14 ± 0.29
Uric acid (mmol/l)^*^	0.36 ± 0.07	0.35 ± 0.08
Energy (kJ/day)	9430 ± 2052	8586 ± 1962
**Prevalence of chronic disease (%)**^+^		
Myocardial infarction	11	15
Stroke	2	6
Diabetes	5	10
Cancer	5	12

^*^Expressed as mean and standard deviation.

^+^The categorical variable chronic diseases (yes/no) had no missing values.

Greatest median increases between baseline and follow-up in food group consumption were observed for fruits and low-fat milk products, whereas the consumption of potatoes, vegetables, high-fat milk products, unhealthy fats and energy free drinks decreased (Table 2). Spearman correlation coefficients between food groups of the two measurement rounds ranged from 0.14 for potato products to 0.71 for strong alcoholic beverages.

**Table 2:** Median (10th , 90th percentile) of the initial 36 defined food groups at baseline and at follow-up in the Zutphen Elderly Study (N = 467)

Food groups (number of aggregated food groups)^*^	**Food consumption (g/d) 1985**	**Food consumption (g/d) 1990**	**Spearman correlation**
**Low-fibre bread (17)**	**20 (0,113)**	**21 (0,100)**	**0.55**
**High-fibre bread (21)**	**99 (0,183)**	**102 (10,188)**	**0.65**
**Low-fibre cereals (28)**	**8 (0,41)**	**10 (0,46)**	**0.26**
**High-fibre cereals (29)**	**0 (0,13)**	**0 (0,23)**	**0.38**
Potatoes (4)	154 (69,286)	125 (63,212)	0.51
Potato products (24)	0 (0,5)	0 (0,11)	0.14
Legumes (31)	0 (0,26)	0 (0,26)	0.28
Vegetables (190)	173 (105,266)	151 (91,234)	0.38
**Fruit (82)**	**143 (26,328)**	**174 (42,361)**	**0.49**
**Fruit juices (23)**	**0 (0,120)**	**0 (0,120)**	**0.31**
**High-fat meat (64)**	**29 (0,61)**	**20 (0,50)**	**0.38**
Low-fat meat (88)	54 (20,102)	56 (23,101)	0.36
Meat products (87)^+^	20 (1,50)	18 (0,47)	0.44
Organ meat (8)	0 (0,5)	0 (0,2)	0.17
**Fatty fish (9)**	**0 (0,18)**	**0 (0,16)**	**0.40**
Lean fish (35)	8 (0,31)	5 (0,29)	0.43
**Egg and egg products (7)**	**16 (2,35)**	**14 (4,31)**	**0.47**
**Cheese (44)**	**29 (8,65)**	**22 (6,59)**	**0.37**
**High-fat milk products (57)**	**109 (0,450)**	**92 (1,418)**	**0.48**
Low-fat milk products (56)	86 (0,470)	161 (0,543)	0.56
Healthy fats (3)^‡^	2 (0,31)	10 (0,40)	0.46
**Unhealthy fats (33)**^‡^	**33 (9,71)**	**22 (6,53)**	**0.50**
Soup (37)	18 (0,114)	9 (0,98)	0.24
Sauce (39)	0 (0,5)	1 (0,8)	0.19
Ready to eat meat snacks (20)	0 (0,1)	0 (0,8)	0.24
**Ready to eat meals (83)**	**0 (0,10)**	**0 (0,28)**	**0.19**
**Sugar and sweets (117)**	**51 (10,109)**	**51 (11,108)**	**0.59**
Cake and biscuits (64)	34 (3,78)	32 (7,71)	0.58
Savoury snacks (13)	0 (0,3)	0 (0,4)	0.34
Nuts and seeds (21)	0 (0,20)	1 (0,13)	0.40
**Energy free beverages (19)**^§^	**856 (500,1375)**	**823 (468,1272)**	**0.54**
**Sugar sweetened beverages (16)**	**0 (0,71)**	**0 (0,101)**	**0.30**
**Beer (3)**	**0 (0,149)**	**0 (0,129)**	**0.52**
**Wine (6)**	**0 (0,50)**	**0 (0,64)**	**0.53**
Light alcoholic beverages (10)^**|**^	0 (0,15)	0 (0,11)	0.54
**Strong alcoholic beverages (11)**^¶^	**10 (0,100)**	**1 (0,70)**	**0.71**

*Food groups included in the final RRR analyses after backwards regression are presented in bold (an extensive list of food grouping can be provided by contacting the corresponding author).

^+^Meat products include products e.g. salami, ham, bacon.

^‡^Healthy fats are defined as fats high in linoleic acid and unhealthy fats as high in saturated fatty acids and trans fatty acids.

^§^Energy free beverages include e.g. water, coffee, tea and light drinks.

|Light alcoholic beverages include e.g. Campari, Port, Sherry, Shandy.

^¶^Strong alcoholic beverages include e.g. Jenever, whiskey, rum, cognac.

Comparison of excluded and included participants showed significant differences for BMI (25.2 kg/m^2^ vs. 25.7 kg/m^2^, p = 0.04) and uric acid (0.37 mmol/l vs. 0.36 mmol/l, p = 0.04). Regarding food group consumption we observed significant differences for cereal products, vegetables, cheese, non-alcoholic and alcoholic drinks, which were all consumed more by included participants, and fats were consumed less by included participants (data not shown).

After backwards regression 19 food groups remained for further RRR analysis. Based on the results of the Van der Voet’s test three dietary patterns were derived from RRR for both examination years. Table 3 shows the three exploratory RRR patterns and percentage of variation explained. Dietary pattern 1 derived in 1985 and 1990 could best be described as the “(low in) cereal fibre pattern”. The characteristics of this pattern were a low intake of high-fibre bread and cereals and a high consumption of fruit juices and sugar sweetened beverages. Pattern 2 was labelled as an “alcohol pattern” showing consistent positive associations with beer wine and strong alcoholic beverages at baseline and follow-up. Pattern 3 did not contain consistent food groups at baseline and after 5 years of follow-up and was therefore labelled “inconsistent”. Percentages in explained variation in single risk factors were slightly different over time, resulting from a slightly different food group composition over the years. The percentage of variation explained in total risk factors and in the 19 food groups were similar over time. The 1st derived RRR pattern explained 6.6% (2nd pattern: 5.6%, 3rd pattern 5.5%) and 6.0% (2nd pattern: 5.9%, 3rd pattern 5.3%) of the variation in dietary variables at baseline and follow-up respectively. The sum of explained variation by all 3 dietary patterns was about 17% in food groups and about 8% in CVD risk factors for both examination years.

**Table 3:** Exploratory RRR dietary pattern weights^* ^**in the Zutphen Elderly Study (N = 467)**

	**RRR dietary pattern weights**^**+**^							
	**Pattern 1 (low in cereal fibre)**		**Pattern 2 (alcohol)**		**Pattern 3 (inconsistent)**			
	**Baseline (1985)**	**Follow-up (1990)**	**Baseline (1985)**	**Follow-up (1990)**	**Baseline (1985)**	**Follow-up (1990)**		
**Food groups**^**+**^								
Low-fibre bread	-0.05	-0.05	-0.09	-0.07	-0.03	0.04		
High-fibre bread	**-0.20**	**-0.27**	0.05	-0.05	0.01	0.09		
Low-fibre cereals	0.08	-0.08	0.02	0.04	**-0.12**	-0.04		
High-fibre cereals	**-0.16**	**-0.25**	0.06	-0.03	-0.02	-0.02		
Fruits	-0.05	-0.04	-0.04	0.03	-0.08	**-0.13**		
Fruit juices	**0.12**	**0.14**	-0.04	-0.02	-0.01	0.03		
High-fat meat	0.00	0.09	-0.03	0.01	0.08	-0.03		
Fatty fish	0.04	**0.11**	-0.04	0.02	**0.13**	0.01		
Egg and egg products	-0.05	0.04	0.04	-0.04	0.07	0.09		
Cheese	**-0.10**	0.03	0.03	0.06	**0.18**	**-0.13**		
High-fat milk products	**-0.12**	-0.08	0.08	**-0.14**	-0.05	**0.10**		
Unhealthy fats	0.00	**-0.17**	0.00	**0.15**	0.01	**-0.12**		
Ready to eat meals	**0.10**	0.00	**0.10**	0.05	0.01	**0.10**		
Energy free beverages	0.07	0.09	-0.04	0.02	-0.06	-0.06		
Sugar and sweets	-0.09	**-0.10**	0.08	0.04	**-0.15**	0.03		
Sugar sweetened beverages	**0.16**	0.06	-0.07	-0.04	-0.07	-0.07		
Beer	**0.14**	**0.12**	**0.12**	**0.11**	-0.05	0.03		
Wine	**0.12**	**0.10**	**0.10**	**0.14**	0.03	0.08		
Strong alcoholic beverages	0.07	-0.05	**0.26**	**0.13**	-0.01	**0.14**		
**% explained variation in**							**Total**^**‡ **^**1985**	**Total**^**‡ **^**1990**
BMI	5.1	12.1	2.8	0.0	2.3	0.5	10.2	12.6
Total cholesterol	2.3	3.5	0.1	1.2	3.4	0.3	5.8	5.0
HDL cholesterol	0.1	2.2	8.6	7.3	1.8	1.1	10.5	10.7
Systolic BP	4.5	1.1	2.0	0.1	0.3	3.7	6.8	4.8
Diastolic BP	4.9	2.9	0.8	0.3	0.7	4.9	6.4	8.2
Uric acid	7.0	4.2	0.3	2.7	1.8	0.1	9.1	7.0
All six risk factors	4.0	4.3	2.5	1.9	1.7	1. 8	8.2	8.0
All nineteen food groups	6.6	6.0	5.6	5.9	5.5	5.3	17.7	17.2

^*^For this analyses we relied on the factor weights (regression coefficient in the RRR model) instead of loadings (correlation coefficient between the food pattern and the food groups) as suggested by Imamura *et al*. [24].

^+^A negative dietary pattern weight reflects a low intake of this food group, whereas a positive dietary pattern weight reflects high intakes of this food group for a person that scores high on the specific dietary pattern.

^‡^Total variation explained in CVD risk factors equals the cumulative percentage in explained variation of all three food patterns derived from RRR.

Table 4 shows significant consistent positive correlations with the “(low in) cereal fibre pattern” for all risk factors except for HDL-cholesterol in 1990. The adherence to the “(low in) cereal fibre pattern” resulted in lower fibre intake from cereals and bread and a higher risk factor profile. The “alcohol pattern” showed significant positive associations with HDL-cholesterol at both time points. Also the “inconsistent pattern” showed a positive association with HDL-cholesterol at baseline and follow-up. However, in contrast to the other two patterns, this association might be caused by different food groups in 1985 and 1990 as the food group weights were different in these years. The confirmatory 1990 and 1985 “low in cereal fibre” dietary patterns showed correlation coefficients with risk factors similar to the exploratory RRR scores (Table 4)

**Table 4:** Pearson correlation coefficients of the food patterns derived from RRR with cardiovascular risk factors and percentage variation in food group intakes and risk factors explained by the food patterns in the Zutphen Elderly Study (N = 467)

	**Pattern 1**		**Pattern 2**		**Pattern 3**		**Pattern 1**		**Pattern 1**	
	(low in cereal fibre)*		(alcohol)*		(inconsistent)*		**(low in cereal fibre)**^**+**^		**(low in cereal fibre)**^**+**^	
	**Baseline (1985)**	**Follow-up (1990)**	**Baseline (1985)**	**Follow-up (1990)**	**Baseline (1985)**	**Follow-up (1990)**	**Confirmatory (1990)**		**Confirmatory (1985)**	
**CVD risk factors**	**1985**	**1990**	**1985**	**1990**	**1985**	**1990**	**1990**	**1985**	**1990**	**1985**
BMI	0.23^‡^	0.35^‡^	-0.17^‡^	0.00	0.15^‡^	-0.07	0.21^‡^	0.21^‡^	0.26^‡^	0.28^‡^
Total cholesterol	0.15^‡^	0.19^‡^	-0.04	0.11^‡^	0.18^‡^	-0.05	0.13^‡^	0.15^‡^	0.11^‡^	0.15^‡^
HDL cholesterol	0.03	-0.15^‡^	0.29^‡^	0.27^‡^	0.14^‡^	0.11^‡^	0.07	0.01	-0.09	-0.09
Systolic BP	0.21^‡^	0.10^‡^	0.14^‡^	-0.03	-0.05	0.19^‡^	0.19^‡^	0.12^‡^	0.10^‡^	0.11^‡^
Diastolic BP	0.22^‡^	0.17^‡^	0.09	-0.06	-0.08	0.22^‡^	0.21^‡^	0.14^‡^	0.09	0.12^‡^
Uric acid	0.26^‡^	0.20^‡^	-0.05	0.16^‡^	-0.13^‡^	-0.03	0.24^‡^	0.24^‡^	0.11^‡^	0.16^‡^

*Exploratory dietary pattern scores were calculated at baseline and follow-up by summing the product of the standardized (mean = 0 and standard deviation = 1) food group weight and the individual’s standardized food group intake (score = ∑ (weight (x) *consumption (x) (g/d)) [24]; For the RRR analysis in 1985 means and standard deviations of that year were used. For Exploratory 1990 means and standard deviations of 1990 were used.

^+^Confirmatory 1990 used standardized dietary intake data of 1990 and weights derived in 1985.Confirmatory 1985 used standardized dietary intake data of 1985 and weights derived in 1990.

^‡^Significant correlation p < 0.05.

Table 5 shows the correlation coefficients between the derived dietary patterns at baseline and follow-up. The strongest correlation between exploratory derived dietary patterns at baseline and follow-up was observed for the “(low-in) cereal fibre pattern”. Confirming the “(low in) cereal fibre pattern” using 1990s diet and 1985 derived RRR weights, showed a slightly stronger correlation with the “(low-in) cereal fibre pattern” derived with loadings and diet of 1985 (0.60 vs. 0.47). Correlation coefficients between confirmatory pattern 1985 and exploratory 1985 showed a correlation coefficient close to 1. The correlation coefficient between the 1985 and 1990 confirmatory scores was slightly lower compared to the correlation coefficient between exploratory patterns derived in 1985 and 1990.

**Table 5:** Pearson correlation coefficients between exploratory and confirmatory dietary patterns in 1985 and 1990 in the Zutphen Elderly Study (N = 467)

**Dietary patterns**	Exploratory* 1990			**Confirmatory**^**+ **^**1990 (diet (D) 1990, loading (L) 1985)**	**Confirmatory 1985 (D: 1985, L: 1990)**
**Exploratory 1985**	**Pattern 1 Low in cereal fibre**	**Pattern 2 alcohol**	**Pattern 3 inconsistent**	**Pattern 1 Low in cereal fibre**	**Pattern 1 Low in cereal fibre**
Pattern 1	0.47^‡^	0.31^‡^	0.05	0.60^‡^	0.73^‡^
Pattern 2	-0.22^‡^	0.34^‡^	0.35^‡^	0.11	-0.41^‡^
Pattern 3	0.08	0.16	-0.04	0.05	0.20^‡^
Confirmatory^+^ 1985 (D: 1985, L: 1990)	0.55^‡^	0.06	-0.09	0.43^‡^	1.00
Confirmatory 1990 (D: 1990, L: 1985)	0.71^‡^	0.40^‡^	0.24^‡^	1.00	0.43^‡^

*See footnote *Table 4.

^+^See footnote ^+^Table 4.

^‡^Significant at p < 0.0001.

Several sensitivity analyses were performed on the first derived pattern as this pattern by definition explains most variation in the chosen risk factors, and is therefore most stable over time. Energy adjustment of the food group intakes as well as the exclusion of BMI from the response variables resulted in similar dietary patterns compared to the initially derived patterns at baseline and follow-up (data not shown). Limiting our population to those who had no chronic diseases (n = 368 participants without myocardial infarction, stroke, diabetes or cancer) at baseline showed a similar correlation coefficient for the exploratory derived “(low in) cereal fibre pattern” between baseline and follow-up (r = 0.50) compared to r = 0.47 in Table 5. Correlation coefficients between dietary patterns derived from RRR using 36 food groups in the full (n = 763) and reduced sample (n = 467) of 1985 were high (correlation coefficient comparing 467 participants of the full and reduced sample r = 0.75). The full study sample of 763 participants showed significant cross validation tests for 36 food groups. Comparability of the patterns derived in the full and reduced sample increased after backwards regression (r =0.86).

## Discussion

Three exploratory dietary patterns were derived from RRR at baseline and at follow-up in a male Dutch elderly population. We labelled the dietary patterns as a “(low in) cereal fibre pattern”, an “alcohol pattern” and an “inconsistent pattern”. The exploratory “(low in) cereal fibre pattern” was relatively stable over time. Stability was represented by a similar pattern structure of high weighing food groups at baseline and follow-up, consistent associations between the derived patterns and CVD risk factors and a moderate correlation coefficient of the “(low in) cereal fibre pattern” between baseline and follow-up.

A trend towards a healthier diet over time was observed by a significant decrease in high-fat meat and meat products and a significant increase in fruit intake, low-fat milk products and healthy fats. Furthermore, we found a decrease in energy intake, as reported by other investigators studying elderly populations [28-31]. Correlation coefficients for the main food groups were comparable to those of the Zutphen Elderly Study obtained after one year of follow-up [32]. This suggests that, in the Zutphen Elderly Study, the relative position of the participants in the distribution of the food groups was relatively stable during follow-up.

The application of RRR has some disadvantages. RRR requires a reasonable sample size for an appropriate examination. Reducing our sample size to 467 participants has likely influenced the non-significant results for the Van der Voet’s test. Furthermore, RRR is linked to two arbitrary choices: 1) the selection of risk factors as response variables and 2) the selection of food groups. We applied known risk factors for CVD available in the Zutphen Elderly Study. Choosing risk factors based on literature is associated with limitations, as these risk factors might not necessarily be highly correlated with each other. Low correlations might result in the derivation of dietary patterns with a low predictability for the chosen risk factors. Bias towards a positive finding related to stability of dietary patterns seems unlikely but the influence of response variables on dietary patterns should be taken into account in studies focussing on the interpretation of dietary patterns.

The decision for backwards elimination of food groups prior to the RRR analysis was taken based on the result of the Van der Voet’s test and was influenced by the approach chosen by Weismayer *et al*. [6] and Newby *et al*. [5]. Both authors applied confirmatory factor analysis, meaning factor analysis was applied twice. After the first run of factor analysis, those food groups with highest factor loadings were selected, on which the second run of factor analysis was applied. As factor loadings represent the bivariate correlation between food groups and derived food patterns, our backwards approach resembles a simplified form of what is called confirmatory factor analysis. Instead of correlation coefficients (equivalent to loadings), regression coefficients (equivalent to weights) were used. Weismayer *et al*. [6] reported that confirmed factor scores were slightly stronger correlated over time (healthy pattern 0.57 vs. 0.63 after 5 years) and Newby *et al*. [5] concluded that confirmatory factor scores were highly correlated with exploratory scores and reproducible over time. Therefore, we assume that our analysis gained in quality by the application of backwards regression. However, bias towards better reproducibility in the Zutphen Elderly Study cannot be excluded as food groups were selected because of good predictability on the selected response variables across the study years 1985 and 1990. Potential bias is expected to be small as only one variable (wine) was included in the set of food groups that was important for 1990 and not 1985. However, ideally one would test the selection of food groups in an independent study sample.

Examining the influence of change in diet (keeping weights constant for 1985 and 1990) increased the correlation coefficients only slightly to 0.60. Changing the weights but keeping the same foods increased the correlation to 0.73. The reason for a correlation of smaller than 1, is likely influenced by changes in food groups and small changes in biomarkers. A slight influence of biomarkers on the stability of dietary patterns was expected, given that changes over time in response variables were only observed in diastolic blood pressure. Furthermore, we ran several sensitivity analyses to examine the influence of the subjective decisions taken. Regarding the risk factors used, we expected BMI to play an important role in the formulation of RRR food patterns, due to the association with CVD [19]. Additional sensitivity analysis showed that dietary patterns remained essentially similar after the exclusion of BMI. This is in line with the results obtained by Schulze *et al*. [33] and indicates that the correlation between BMI and food groups on one hand and BMI and the chosen risk factors on the other hand, did not corrupt the pattern structure and did not influence the stability of the patterns.

Additional energy adjustment on predictor variables did not change the dietary pattern structure in our population. The reason for this could be the homogeneous character of the Zutphen Elderly population regarding energy intake, age and sex. After energy adjustment, Kröger *et al*. [34] found a decrease of about 15 percentage points in total explained nutrient variation by the first pattern derived from RRR. Our results for the percentage in explained CVD risk factor variation was similar before and after energy adjustment. Whether energy adjustment should be performed depends on the research question and on the population under study [23,24,35].

For this study we lost 50% of the participants from the initial baseline sample, as we wanted to measure the same group of people at two different time points. Two reasons were responsible for the loss of participants. Men dying between 1985 and 1990 and men non-responding (22%) at the follow-up examination, which might result in a “more healthy” population in comparison to the general Dutch population. However, we do not consider the selection of healthy elderly participants or the information on dietary intake data used from two decades ago as a major limitation. The current manuscript focussed on the methodology of RRR and the potential of RRR to derive stable dietary patterns over time. The advantage of the present study was the assessment of diet by a cross-check dietary history method at both examination years. A reproducibility study on the performance of the cross-check dietary history method examined in the Zutphen Study revealed that measurement error of the cross-check dietary history method was small [32]. Therefore, we assume that correlations between food groups and response variables in the present study were slightly underestimated and affected the dietary patterns derived from RRR only marginally.

In conclusion, the results of the present study on the stability of dietary patterns are in accordance with those reported in the literature. The “(low in) cereal fibre pattern” was the most stable pattern especially in apparently healthy elderly men. RRR analysis remains an attractive approach for nutritional epidemiology and the validity of this pattern should be further evaluated in subsequent diet-disease analyses.

## Competing interests

The authors declare that they have no competing interests.

## Authors’ contributions

The contributions of the authors were as follows: DK provided the data of this study. EK, CdG, EF, MS, SS and NJ were responsible for the study concept and design. EF, MS and NJ take the responsibility for the integrity of the data and the accuracy of the data analyses and drafting of the manuscript. The statistical analyses were performed by NJ, HB and MS advised on the statistical analyses procedure. All authors contributed to the intellectual content of the manuscript and critically reviewed the different versions. All authors read and approved the final manuscript.

## Supplementary Material

Additional file 1: Table S1 Pearson correlation coefficients between CVD risk factors of 1985 and 1990 in the Zutphen Elderly Study (N = 467). Click here for file
