# Supplementary material for: Stability of dietary patterns assessed with reduced rank regression; the Zutphen Elderly Study
Source: Nutr J. 2014 Apr 1;13:30. doi: 10.1186/1475-2891-13-30 (PMC4021363; doi:10.1186/1475-2891-13-30)
Supplement: Additional file 1: Table S1 — Pearson correlation coefficients between CVD risk factors of 1985 and 1990 in the Zutphen Elderly Study (N = 467). [file 1475-2891-13-30-S1.docx]

| Supplementary Table 1. Pearson correlation coefficients between CVD risk factors of 1985 and 1990 in the Zutphen Elderly Study (N=467) | | | | | | | |
| --- | --- | --- | --- | --- | --- | --- | --- |
| 1985 |  | 1990 | | | | | |
| Response variables |  | BMI | Total cholesterol | HDL cholesterol | Systolic BP | Diastolic BP | Uric acid |
| BMI |  | 0.87* | 0.12* | -0.26* | 0.15* | 0.29* | 0.16* |
| Total cholesterol |  | 0.13* | 0.78* | 0.08 | 0.07 | 0.05 | 0.11* |
| HDL cholesterol |  | -0.22* | 0.12* | 0.79* | 0.03 | -0.06 | -0.22* |
| Systolic BP |  | 0.07 | 0.03 | 0.06 | 0.62* | 0.63* | -0.05 |
| Diastolic BP |  | 0.23* | 0.04 | 0.02 | 0.63* | 0.53* | 0.04 |
| Uric acid |  | 0.18* | 0.11* | -0.21* | 0.05 | 0.12* | 0.68* |

*Significant at p<0.05
